# A method for measuring meaningful physiological variables in fish blood without surgical cannulation

**DOI:** 10.1038/s41598-023-28061-w

**Published:** 2023-01-17

**Authors:** William George. Davison, Christopher A. Cooper, Katherine A. Sloman, Rod W. Wilson

**Affiliations:** 1 Biosciences Department, College of Life and Environmental Sciences, University of Exeter, Exeter, UK; 2 International Zinc Association, Avenue de Tervueren 168, 1150 Brussels, Belgium; 3 Institute for Biomedical and Environmental Health Research, University of the West of Scotland, Paisley, UK

**Keywords:** Haemic and immune systems, Animal physiology, Ichthyology

## Abstract

Gaining meaningful blood samples from water-breathing fish is a significant challenge. Two main methods typically used are grab ‘n’ stab and surgical cannulation. Both methods have benefits, but also significant limitations under various scenarios. Here we present a method of blood sampling laboratory fish involving gradual induction of anaesthesia within their home tank, avoiding physical struggling associated with capture, followed by rapid transfer to a gill irrigation system to maintain artificial ventilation via adequate gill water flow and then followed by sampling the caudal vasculature. This method negates many blood chemistry disturbances associated with grab ‘n’ stab (i.e., low pH and oxygen, elevated lactate, CO_2_ and stress hormones) and generates results that are directly comparable to cannulated fish under a wide range of experimentally-induced acid–base scenarios (acidosis and alkalosis). Crucially this method was successful in achieving accurate acid–base blood measurements from fish ten times smaller than are typically suitable for cannulation. This opens opportunities not previously possible for studies that relate to basic physiology, sustainable aquaculture, ecotoxicology, conservation, and climate change.

## Introduction

Measuring blood chemistry variables is a key element of research to investigate fundamental physiological mechanisms and understand how fish respond to natural environmental variability and the challenges presented by anthropogenic change (e.g. pollution, climate change, aquaculture scenarios etc.). Valid blood chemistry data can be vital in revealing the health and performance of individuals, populations and entire ecosystems. In fish, blood parameters have been measured to inform a wide range of physiological purposes such as haematology, biochemistry, immunology, reproduction, nutrition, ecotoxicology and wider health status. While a variety of methods are available for obtaining blood samples in fish^1^ concerns over practicality and accuracy have led to the prominence of two methods. The colloquially named ‘grab n’ stab’ technique involves rapidly capturing and removing a fish from the water and then rendering it insensible (either through anaesthesia or stunning/killing) before blood sampling^1,2^. The second is through prior surgical implantation of an indwelling cannula into a blood vessel (often the dorsal aorta, but also a caudal or branchial artery/vein) followed by return to water and recovery from anaesthesia and surgery after which serial blood samples can be taken via the cannula with minimal disturbance while the fish remains submerged^2–4^.

**Figure 1:**
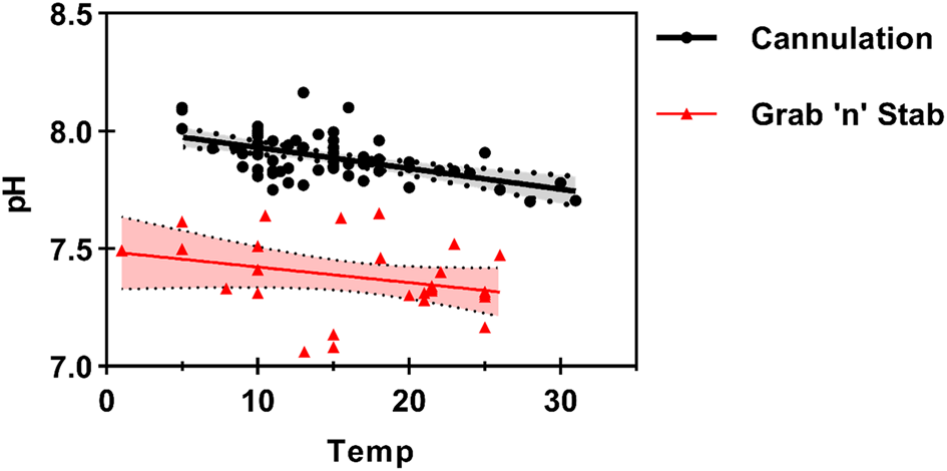
The relationship between blood pH and temperature for a range of fish species. Black filled circles represent data obtained from surgically cannulated fish from 38 species in total (based on the review by Hillman & Hedrick^23^ with much of the original data collated by Ultsch & Jackson^24^). Red triangles represent data collected using the grab n’ stab method from a range of species collated from published papers by the current authors (see Supplementary Material). Solid lines represent regression relationships through the data collected via the two blood sampling methods. The dotted lines around the regression line are the 95% confidence intervals. Linear regression analysis showed that the slopes of the two lines were not significantly different (p = 0.58), but the elevations were significantly different from each other (p < 0.0001), with the average difference between the two methods being 0.53 pH units, which represents a threefold increase in H^+^ ions using grab ‘n’ stab.

The ‘grab ‘n’ stab’ technique is much simpler to perform, especially under field conditions, and if performed correctly it involves minimal risk of harm to the animal if anaesthesia and full recovery prior to release are employed. Thus, it has been used extensively to characterise the blood parameters of ‘wild’ fish and in many laboratory studies^5–10^. If blood samples are obtained quickly enough after capture, the ‘grab ‘n’ stab’ technique can also yield comparable results to cannulation in relation to several hormonal and ionic blood parameters^11^. However, some variables are particularly prone to rapid change induced by the grab n’ stab procedure (e.g. blood gases and acid–base variables, haematology and stress hormones), which presents a significant challenge for valid blood sampling of water-breathing fish. There are two main reasons for this. Firstly, more than half of the body mass in fish consists of anaerobic white muscle^12^ which rapidly generates large amounts of metabolic acid during the powerful burst swimming activity during the escape response initiated by the capture process^7,10,13–16^. Secondly, the gills collapse^15^ and bradycardia is induced^17^ when fish are removed from water preventing respiratory gas exchange and causing a steady rise in excretory metabolites (e.g. CO_2_, H^+^, lactate, ammonia, urea) and a corresponding fall in pH and O_2_ in proportion to the duration of air-exposure. In addition, the blood buffering capacity is 3 to 10 times lower in fish than in terrestrial vertebrates due to 5–10 times lower plasma bicarbonate levels and substantially lower non-bicarbonate buffering (due to fewer red blood cells and therefore haemoglobin^18^, so the same amount of acid addition causes a much larger pH shift). Simultaneous release of the catecholamine ‘stress’ hormones also causes two further perturbations. Firstly, they stimulate proton extrusion out of the red blood cells of fish leading to further plasma acidification^19,20^. Secondly, they stimulate a release into circulation of red blood cells stored in the spleen and a swelling of individual red blood cells, which collectively cause haematocrit to rise^21^. Wood et al*.*^22^ went as far as to describe blood gas and acid–base data obtained using the grab n’ stab method as essentially “meaningless”. Our analysis of previously published data in Fig. 1 demonstrates this very clearly by showing that across a ~ 30 °C temperature range and 37 published studies, the blood pH obtained from the grab n’ stab method is approximately 0.5 pH units lower than from cannulated fish (i.e. a threefold difference in terms of free H^+^ ion concentration across this temperature range).

Where ‘grab ‘n’ stab’ is not appropriate, experiments assessing more labile blood chemistry variables have utilised surgical cannulation. Fish are held under anaesthesia while a chronically indwelling cannula is implanted into an artery or vein (most commonly the dorsal aorta) before a period of recovery^4^ (typically between 24 and 72 h as this allows circulating catecholamines to return to resting values^25^). Cannulation has been used to successfully sample a variety of blood vessels (reviewed by Axelsson & Fritsche^3^ and Iwama & Ishimatsu^26^). In a few cases more sophisticated cannulation of multiple vessels has been used to continuously monitor blood variables using an extracorporeal loop to shunt blood past various real-time sensors before returning it to the fish^27–29^. Importantly, cannulation can therefore be used to remove blood samples via the cannula while the fish is still submerged and breathing water, and without the acute stress associated with capture during the ‘grab n’ stab’ method. However, the surgical implantation of a cannula instead provides a level of chronic stress to the animal. In some species another stress hormone, cortisol, can be elevated for up to a week post-surgery^30,31^ and immune function impaired for up to two weeks post-surgery^31^. Cannulation is also constrained by the need for fish to be of sufficient size for surgery to be feasible (typically > 150 g) and therefore cannot be applied to small fish. An issue also arises due to the need to keep fish isolated after cannulation to avoid accidental removal of cannulae due to social activity. For many fish, social isolation in itself is sufficient to elicit a stress response^32^. Another factor relevant to many studies is that cannulated fish seldom resume normal feeding behaviour (or sometimes do not feed at all) before cannulae become blocked and lose their patency.

It would therefore be desirable to have a method that avoids the acute stress of the grab n’ stab approach, as well as the technical difficulties and chronic stresses of surgical cannulation. With this in mind, we tested the efficacy of a novel method for blood sampling (termed *in-situ* anaesthesia) that avoids both these challenges and can provide an accurate determination of blood gas and acid–base variables with the added advantage it can be used without impairing normal behavioural processes such as feeding. This method involves induction of anaesthesia within the experimental tank before transfer to a gill irrigation table that can maintain normal ventilation while sampling the caudal vasculature. Validation of this method involved three stages: firstly the novel method was compared to two variations of the grab ‘n’ stab method (one with no ventilation, and one with artificial gill ventilation); secondly, the novel method was compared to paired samples in cannulated fish (i.e. samples were taken from indwelling canulae and then the same fish sampled using *in-situ* anaesthesia); and finally, the novel method was performed on fish typically too small to be cannulated.

## Methods

### Ethical approval

All experiments were conducted in accordance with the UK Animals (Scientific Procedures) Act 1986 and completed under a UK Home Office project license (Experiment 1 &3: PPL-P88687E07, and Experiment 2: PPL-30/2217). Work was also performed with the approval of the University of Exeter Animal Welfare and Ethical Review Body (AWERB). Where possible, all work has been carried out and reported in accordance with ARRIVE guidelines (See Supplementary Materials). Sample sizes determined based on variation observed in previous experimental work on trout in the lab group.

### Fish husbandry

All-female diploid rainbow trout (*Oncorhynchus mykiss*) were obtained from Houghton Springs Fish Farm (Houghton, Dorset, UK) and housed at the University of Exeter. Before experimentation fish were housed in 300–400 l tanks fed with dechlorinated Exeter freshwater at 11 °C (Experiment 2) or 15 °C (Experiments 1 and 3). Where necessary fish had food withheld for a minimum of seven days to allow full gut clearance ahead of blood sampling.

### Experiment 1: comparison of fish handling methods on blood sampling

The aim of experiment 1 was to assess the impact of different blood sampling methods on acid–base, haematological, and stress biomarkers of fish held in ‘control’ water chemistry. This will compare the novel *in-situ* anaesthesia blood sampling method with two variations on grab ‘n’ stab sampling (termed “Stock Tank/Netted” and “Isolated/Netted”).

Before experiments, all fish (n = 20, mean body mass ± SEM = 195.6 ± 6.13 g) were haphazardly allocated to one of three treatment groups which were based on the degree of acute disturbance the fish received prior to taking a caudal vessel blood sample. These treatments are referred to as (1) “Stock Tank/Netted”, (2) “Isolated/Netted” and (3) “Isolated/in-situ Anaesthesia”. The “Stock Tank/Netted” treatment is intended to represent a variation of grab ‘n’ stab sampling where no effort has been made to minimise or account for the acute physical stress of sampling. The “Isolated/Netted” treatment is intended to represent a variation of grab ‘n’ stab sampling where some effort has been made to offset the impacts of capture stress on blood parameters. All ‘Isolated’ fish in the second two groups were transferred from the main stock tank to 25-l isolation tanks and allowed to acclimate for a minimum of 24 h and a maximum of 5 days to recover from the initial netting and handling during transfer from the stock tank. Each 25 l tank was supplied with dechlorinated freshwater and aerated by a diffuser in the tank.

The “Stock Tank/Netted” fish (n = 8) were individually netted directly from the stock tank (volume =  ~ 500 l) which housed approximately 15 fish together (only eight sampled), and transferred to a 10 l bucket containing a knockout dose of 75 mg/l of benzocaine until equilibrium was lost and fish no longer responded to a fin pinch. Fish were then removed from water and sampled in air on a bench by caudal puncture with an ice-chilled heparinised 1 ml syringe (23G needle) before termination by the destruction of the brain.

The “Isolated/Netted” fish (n = 6) were isolated, as described above, before blood sampling to avoid the acute handling disturbance experienced by the “Stock Tank/Netted” group immediately prior to their own sampling. At the point of blood sampling, these “Isolated/Netted” fish were rapidly netted from the isolation tank and transferred to a 10 l bucket containing a knockout dose of 75 mg/l of benzocaine until fish lost equilibrium and did not respond to a fin pinch but were still ventilating. Fish were then immediately transferred to an independent gill irrigation system (described in supplementary materials) containing a maintenance dose of 30 mg/l of benzocaine. The fish were supported on their backs in a v-shaped sponge that lightly held the opercula in a just-closed position while keeping the entire gill basket submerged and elevating the ventral surface between the anal and caudal fins just above the waterline. A small pump with an adjustable clamp on the output tube was used to irrigate the gills at ~ 400 ml/kg/min (approximately twice the normal ventilation volume^33^) sufficient to separate the branchiostegal membrane from the body wall so that a small but distinct flow of water through the gills was just visible. Ventilatory rates above 400 ml/kg/min will have a negligible effect on arterial blood gas tensions^34^ providing a useful safety-margin. However, ventilating below 400 ml/kg/min, or having the gill basket above water level risks impairing gas exchange, risks inducing hypoxia and elevating blood pCO_2_. Once appropriate ventilation had been achieved, the fish were blood sampled by caudal puncture with ice-chilled heparinised syringes before termination by the destruction of the brain.

The “Isolated/*in-situ* anaesthesia” fish (n = 6) had water flow to their individual tank ceased before sampling. A siphon was used to remove 2 l of water from the tank with 1 l being retained for preparing a stock anaesthetic solution of 1.875 g/l benzocaine by diluting a stock solution of 100 g/l benzocaine in ethanol. This mixture was then slowly introduced over 1 min without disturbing the fish (via a funnel placed through a hole in the lid and above the air stone to maximise mixing) to give a knockout dose of 75 mg/l in the individual fish chamber. The fish was then left until equilibrium was lost and the fish did not react to a fin pinch but was still lightly ventilating (~ 2 min). Fish were then transferred to the gill irrigation table and blood sampled from a caudal vessel as described above.

### Analysis of blood and plasma

For the endpoints measured herein, no blinding was performed as bias is avoided using protocols that provide an objective output from automated analytical instruments. After sampling blood was immediately analysed for a range of blood chemistry and haematology endpoints. Blood PO_2_ was measured on whole blood in a thermostatically controlled system set to experimental temperature (Strathkelvin 1302 electrode and 781 m; Strathkelvin Instruments Ltd, Glasgow, UK). Whole Blood extracellular pH (pHe) was measured on 30 µl of whole blood using an Accumet Micro pH electrode and Hanna HI8314 membrane pH meter at 15 °C calibrated to pHNBS 7.04 and 4.02 specific buffers at 15 °C. Three 75 µl microcapillary tubes were filled with whole blood and sealed with Critoseal capillary tube sealant (Fisher) and paraffin oil. These were then centrifuged for two minutes (Hawksley Micro-haematocrit Centrifuge; 10,000 RPM for 2 min) and haematocrit measured using a Hawksley micro-haematocrit reader. Plasma was then extracted from these tubes and analysed for TCO_2_ using a Mettler Toledo 965D carbon dioxide analyser. Plasma PCO_2_ and [HCO_3_^−^] were then calculated from TCO_2_, temperature and blood pH using the Henderson-Hasselbalch equation using values for solubility and pK_app_ based on Boutilier et al.^35^. The haemoglobin content of the blood was also assessed by the cyanmethemoglobin method (using Drabkin’s reagent, Sigma). Mean corpuscular haemoglobin content (MCHC) was then calculated using the equation:

$$\text{MCHC = }\frac{\text{[Hb]}}{\text{Hct}} \times 100$$

Any remaining whole blood was then centrifuged at 10,000 rpm for 2 min at 4 °C prior to further analysis. A 100 µl aliquot of plasma was separated and snap-frozen in liquid nitrogen before analysis for glucose and lactate using a YSI 2900D Biochemistry Analyser (Xylem Analytics, UK). A further 20 µl aliquot of plasma was diluted into ultra-pure water before analysis of ions using a Dionex ICS-1000 and ICS-1100 ion chromatography system. A further 200 µl aliquot was mixed with 15 µl of 0.2 M reduced glutathione antioxidant before snap freezing in liquid nitrogen. These were later analysed for cortisol, noradrenaline and adrenaline using commercial ELISA kits (Cortisol = Enzo Life Sciences, ADI-900-071. Noradrenaline and adrenaline = ABNova, KA1877). The remaining red cell pellet had any remaining plasma and white cells removed before snap freezing in liquid nitrogen. It was then later analysed for pH_i_ using the freeze-and-thaw method described by Zeidler and Kim^36^ and validated for use in fish by Baker et al.^37^.

### Experiment 2: comparison of gill irrigation versus cannulation technique

The aim of experiment 2 was to compare blood chemistry of blood samples taken from dorsal aorta cannulas and *in-situ* anaesthesia caudal puncture from the same fish across a variety of different water chemistries (control, acidosis, and alkalosis). These exposures therefore generate three distinct acid–base states within the animal and allow direct comparison of the two methods’ ability to achieve accurate acid–base data across a range of scenarios.

Fish used in Experiment 2 (n = 5, mean body mass 235.0 ± 15.7 g) were not fed for the 7 days prior to experimentation. To allow repetitive blood sampling without disturbance fish were surgically fitted with dorsal aortic catheters (ID 0.58 mm OD 0.96 mm; Portex) under anaesthesia with pH-adjusted MS222 (60 mg l^−1^; Sigma) as described in Cooper & Wilson^38^ (See note in Supplementary Materials regarding anaesthetic choice). Following surgery, fish were transferred to individual 25 l chambers supplied with both aeration and inflow of clean freshwater and allowed a 72-h recovery period before experimental treatments.

On the first day following the 72-h recovery from surgery, a control blood sample was withdrawn from the dorsal aorta catheter, and the blood sample processed for blood gas and acid–base variables (as above). One hour later, the same fish was anaesthetised *in-situ* without prior disturbance using the same *in-situ* anaesthesia method described in Experiment 1. However, in order to validate this method for use with different anaesthetics, in this instance, anaesthesia was achieved using a concentrated stock solution of MS222 (2 g/l). Due to its acidic nature, it was pH adjusted using NaOH to the same pH as measured in the individual fish tank (without any aeration of the stock solution during this preparation). This MS222 stock solution was diluted to the final knockout dose of 80 mg/l in the isolation tank without altering the pH of the water. Once the fish was sufficiently anaesthetised (loss of the righting reflex and lack of a tail pinch reflex) it was kept submerged in the same chamber but transferred into a custom-made sling with the ventral surface between the anal and caudal fins slightly raised just above the water surface, but the head and gills completely submerged. The gills were then irrigated, and blood sampled as described before. Light pressure was then applied around the blood sampling site until it was confirmed that no bleeding would occur. The blood sample was then analysed using an identical methodology to the sample obtained from the dorsal aortic catheter. The water in the chamber was then quickly flushed to waste to remove the anaesthetic and the gill irrigation continued until the fish recovered.

On the next day, the same fish sampled previously were exposed within the same chamber to elevated CO_2_ (nominally 1%; 10,000 µatm). This was achieved by supplying a gas mixture of CO_2_ and air (Aalborg GF17 mass flow controller) to the diffuser within the fish chamber, and recirculating the water exiting the chamber via a 100 l reservoir (which was also aerated with the same gas mixture). This high environmental CO_2_ exposure was maintained for 24 h, at which point a blood sample was first taken via the dorsal aortic catheter, then 1 h later using the gill irrigation technique, as described for the control blood samples using the same methods. Whilst recovering the fish from anaesthetic the water chemistry was returned to control conditions (i.e. using air-equilibrated water with ambient CO_2_) and the fish left for 24 h to recover from the high CO_2_ exposure and anaesthetic.

On the final day of experiments, the fish were then exposed to alkaline water (pH 9.6) using the addition of KOH and glycine buffer achieving a final concentration of 0.75 mM (as used in McGeer & Eddy^39^). Fish were maintained under this condition for 6 h before taking two blood samples as previously described (the first via the dorsal aortic catheter, the second 1 h later using the gill irrigation technique).

### Experiment 3: gill irrigation of fish too small for cannulation

The aim of Experiment 3 was to test whether the *in-situ* anaesthesia/gill irrigation technique is capable of achieving similar blood gas and acid–base status under similar scenarios to those achieved in Experiment 2 (i.e. control, acidosis and alkalosis) in fish that would be otherwise too small to cannulate.

Fish between 18 and 61 g (n = 30, mean body mass 34.39 ± 2.07 g) were removed from stock tanks and allocated to one of three treatment groups referred to as the control group, the high CO_2_ group, and the fed group. Due to space limitations, no randomisation of allocations was feasible, and each treatment group was isolated in sequence (control first, high CO_2_ second, and fed last). Before experiments fish were transferred into individual 25 l isolation tanks supplied with dechlorinated freshwater from a 480 l sump in which water chemistry could be manipulated prior to flowing into the individual fish isolation tanks.

The control fish (n = 10) were transferred into isolation tanks and allowed 24 h to acclimate before being anaesthetised (75 mg/l benzocaine), gill irrigated, and blood sampled as described in Experiment 1. Due to the limited blood volumes of these smaller fish, not all endpoints were analysed as previously. The endpoints analysed were PO_2_, total CO_2_, blood pH, haemoglobin concentration, and haematocrit.

The High CO_2_ treatment group (n = 10) were transferred into isolation tanks and allowed 24 h to acclimatise before CO_2_ dosing was begun. CO_2_ was elevated to nominally 10,000 µatm by bubbling with CO_2_ gas into the sump tank under the control of a solenoid valve until the appropriate pH was reached, controlled by an Aqua Medic pH computer (Aqua Medic, Germany). Fish were then left for 5 days in order to allow full acid–base compensation to occur prior to sampling. The PCO_2_ of the gill irrigation system was also matched to the PCO_2_ of the experimental system by bubbling with a CO_2_-air mix of the correct PCO_2_ using a mass flow controller system (Aalborg, USA). Blood was then sampled and analysed as above.

The fed group (n = 10) was isolated two weeks before sampling to allow time for fish to acclimate to feeding on a 5% ration once per week of commercial pellets (Horizon 100, 4.5 mm 40% protein pellets, Skretting, UK). This feeding induces a blood alkalosis consistent with previous observations of the alkaline tide in rainbow trout^38^ which is a metabolic alkalosis (i.e. caused by elevated HCO_3_^−^) as opposed to a respiratory alkalosis (i.e. caused by reduction in PCO_2_) observed under the high pH treatment in Experiment 2. Fish were anaesthetised *in-situ* 24 h after feeding, gill irrigated, blood sampled and analysed as described below.

### Statistical analysis

All data for Experiment 1 are given as means ± standard error of means and analysis performed using GraphPad Prism version 8.0.0 for Windows, GraphPad Software, San Diego, California USA, www.graphpad.com. Outliers were defined a priori as data falling outside the 95% confidence interval and were excluded from analysis (Details of data exclusions given in Supplementary Materials). Data were tested for the similarity of standard deviations (SD) using Brown-Forsythe tests with a p-value below 0.05 signifying statistically similar SDs. Data that passed the Brown-Forsythe test (blood pH, RBC pHi, plasma pCO_2_, plasma [HCO_3_^−^], plasma [H^+^], [Hb], MCHC, plasma [lactate], plasma [glucose], and plasma [cortisol]) were analysed using an ordinary one-way ANOVA with Tukey’s multiple comparisons test and significance given as p ≤ 0.05. Data that failed the normality test (plasma PO_2_, plasma [adrenaline], and plasma [noradrenaline]) were appropriately transformed (transformations are given in Supplementary Materials) before analysis with one-way ANOVA with Tukey’s multiple comparisons as before.

All data from Experiment 2 are given as presented and analysed using the same software as above for Experiment 1. Outliers were defined a priori as data falling outside the 95% confidence interval and were excluded from analysis (Details of data exclusions given in supplementary materials). Data are presented as both violin plots and linear regressions of caudal puncture samples versus dorsal aorta samples for ease of understanding. Violin plot data were analysed using a repeated measures design of a partition sum of squares general linear model (GLM) with Šídák’s multiple comparisons tests. The linear regression analysis was performed using a simple linear regression comparing data to an equivalents line with alpha designated as p ≤ 0.05.

All data for Experiment 3 are given as mean ± SEM and analysed in the same manner as above. Outliers were defined a priori as data falling outside the 95% confidence interval and were excluded from analysis (Details of data exclusions given in supplementary materials). Data for PO_2_, [Hb], HCt, MCHC, [lactate], and [glucose] passed the normality test while data for pH, [HCO_3_^−^], PCO_2_, and [H^+^] failed. Where data failed normality testing, appropriate data transformations were applied before analysis (Detailed in supplementary materials). However, transformation did not standardise SDs for the PCO_2_ data and therefore a non-parametric Kruskal–Wallis was performed with Dunn’s multiple comparisons test.

## Results

### Experiment 1

**Figure 2:**
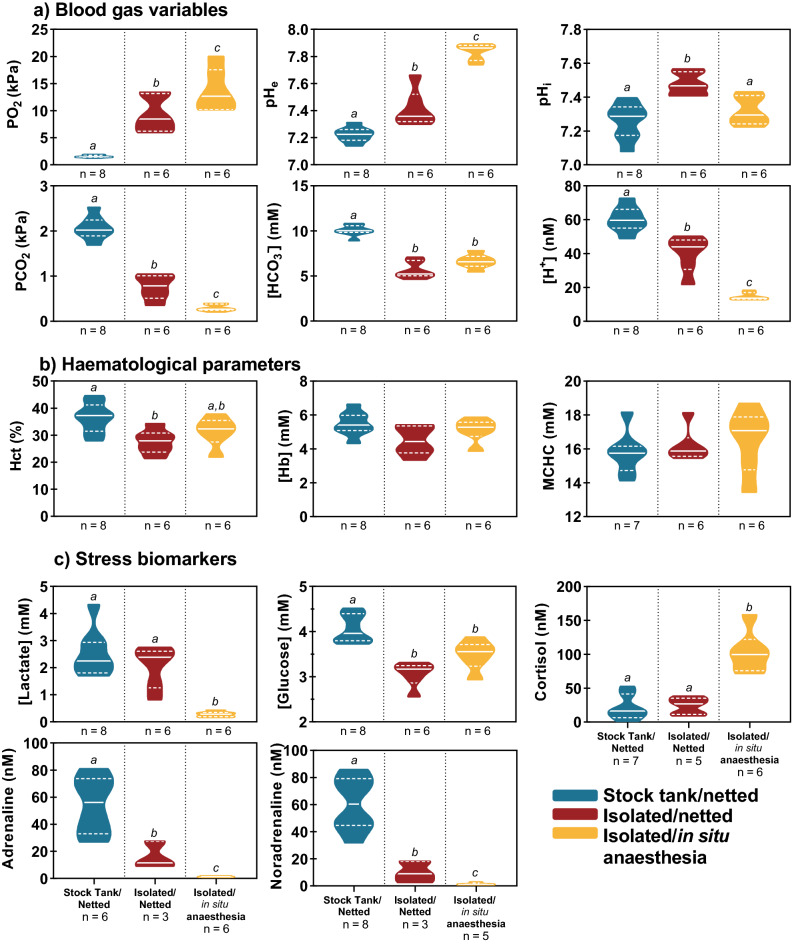
Experiment 1 (**a**) Effect of sampling method on blood gas variables (blood PO_2_, blood pH_e_, RBC pH_i_, blood pCO_2_, plasma bicarbonate concentration, plasma **H**^**+**^ concentration. (**b**) Effect of sampling method on haematological parameters (Haematocrit, haemoglobin concentration & mean corpuscular haemoglobin concentration). (**c**) Effect of sampling method on stress biomarkers (plasma lactate concentration, plasma glucose concentration, plasma cortisol concentration, plasma adrenaline concentration, plasma norepinephrine concentration). Data presented as truncated violin plots with a solid white line denoting the median and dotted white lines indicating the bounds of the inter-quartile range (IQR) and the width of the bar estimating the density of data points using kernel density estimation. Significant differences are denoted by different letters as determined by ordinary one-way ANOVA with Tukey’s multiple comparisons and alpha defined as 0.05.

Concerning the blood gas and acid–base variables (Fig. 2a), sampling methodology significantly affected all parameters (blood PO_2_, pH_e_, PCO_2_ and [H^+^]). In the stock tank/netted group this presented as severe hypoxia, hypercapnia and acidosis. The isolated/netted group experienced mild hypoxia, hypercapnia and acidosis and the isolated/*in-situ* anaesthesia group had PO_2_, PCO_2_ and pH_e_ values that would be normal for arterial blood in cannulated fish^38,40^. Data for red blood cell intracellular pH (pH_i_) showed no difference between stock tank/netted fish and isolated/*in-situ* anaesthesia fish but a significant alkalosis was observed in the isolated/netted fish compared to the other two groups. Plasma bicarbonate was also significantly higher in the stock tank/netted fish compared to either the isolated/netted or isolated/*in-situ* anaesthesia groups which were statistically indistinguishable.

For haematological parameters (Fig. 2b), only haematocrit was impacted by sampling methodology. The stock tank/netted group had significantly elevated haematocrit compared to the isolated/netted fish but not isolated/*in-situ* anaesthesia fish. However, no difference was observed between the isolated/*in-situ* anaesthesia group and either of the other two groups. All stress biomarkers (Fig. 2c) were also highly impacted by sampling methodology. The isolated/*in-situ* anaesthesia fish had the lowest plasma lactate and catecholamine levels (adrenaline and noradrenaline) but had the highest plasma cortisol levels, about 3 times greater than the other two treatments. Conversely, the stock tank/netted fish had the highest catecholamine levels (both adrenaline and noradrenaline), and plasma glucose, which were all significantly greater than the other two groups. Whilst the stock tank/netted fish also had the highest mean lactate levels, this was similar to the isolated/netted group, and both were significantly greater (by about tenfold) than the isolated/*in-situ* anaesthesia group.

### Experiment 2

**Figure 3:**
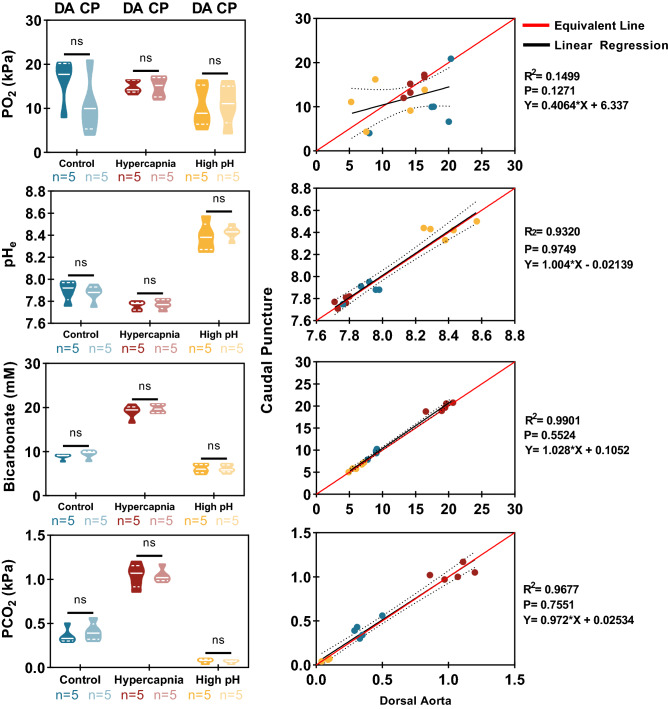
Experiment 2 Effect of three different environmental conditions (control, high CO2 or high pH) on variables of blood sampled either via dorsal aorta cannula (DA, darker violin plots) or paired samples via caudal puncture using in-situ anaesthesia (CP, lighter violin plots). Data are presented as truncated violin plots for each variable versus the three environmental conditions with a solid white line denoting the median and dotted white lines indicating the bounds of the inter-quartile range (IQR) (left panels) or as linear regressions of the caudal puncture versus dorsal aorta sample techniques for each variable (right panels). Significant differences in violin plots are denoted by different letters as determined by a repeated measures design of a partition sum of squares general linear model (GLM) with Šídák’s multiple comparisons tests and alpha defined as 0.05. Linear regressions compared caudal puncture data versus dorsal aorta cannula data (black line ± 95% CI boundaries in black dotted lines) to an equivalence line (red line) where alpha = 0.05.

As intended, the high environmental CO_2_ treatment (Fig. 3) induced a mild, and almost completely compensated, respiratory acidosis (~ 0.15 pH units) with plasma bicarbonate approximately doubling. The high environmental pH treatment induced a strong respiratory alkalosis (+ 0.5 pH units) with plasma bicarbonate dropping slightly but pCO_2_ being about sixfold lower than the control treatment. There was a strong correlation between the two sampling methods (cannulation versus *in-situ* anaesthesia) across all water chemistry treatments (Fig. 3). Plasma PO_2_ values were not found to be different across the environmental exposures or the sampling methodologies. However, linear regressions found that the slope was significantly different from the equivalence line. In contrast, the acid–base relevant measurements (pHe, PCO_2_, and [HCO_3_^−^]) were found to all be significantly different from each other across all exposures, but not between sampling methods with linear regression analysis confirming this in all cases.

### Experiment 3

**Figure 4:**
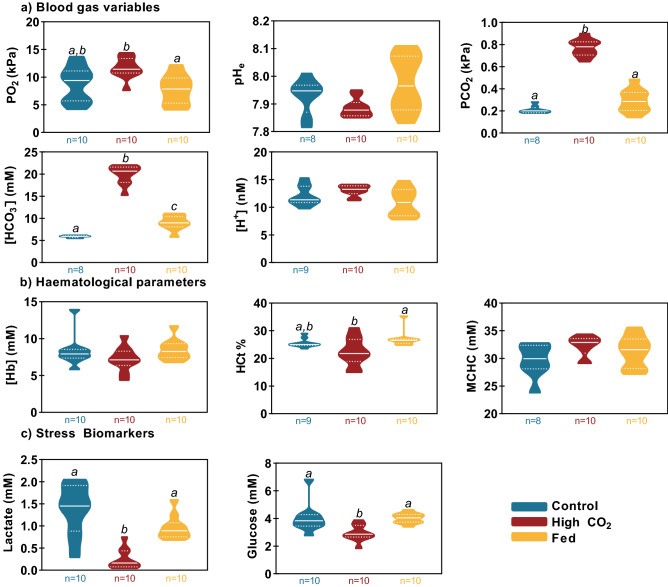
Experiment 3 Effect of high environmental CO_2_ and feeding on blood gas variables in trout below cannulatable size. (**a**) Blood gas variables (blood PO_2_, blood pH_e_, blood pCO_2_, plasma bicarbonate concentration). (**b**) Haematological parameters (haemoglobin concentration, haematocrit, mean corpuscular haemoglobin concentration). (**c**) Stress biomarkers (lactate concentration, glucose concentration) Data presented as truncated violin plots with a solid white line denoting the median and dotted white lines indicating the bounds of the inter-quartile range (IQR). Significant differences are denoted by different letters as determined by ordinary one-way ANOVA with Tukey’s multiple comparisons and alpha defined as 0.05.

When sampling the smaller fish (below cannulation size) using the *in-situ* anaesthesia approach, the different treatments induced the intended changes in blood acid–base balance (Fig. 4a). Specifically, the high environmental CO_2_ treatment induced a compensated respiratory acidosis with no significant change in blood pH, and plasma bicarbonate rising significantly by ~ fourfold to offset a similar fold-rise in pCO_2_. Feeding induced a significant 52% rise in plasma bicarbonate with no change in pCO_2_ as expected, but blood pH was not significantly higher than the control treatment. Blood PO_2_ was significantly higher in the fish exposed to high CO_2_ compared to the fed fish with no other differences observed.

Measurements of haematological parameters (Fig. 4b) were largely unaffected by different treatments with the only differences observed in haematocrit which was slightly lower in the high CO_2_ group compared to the fed group.

Values of stress biomarkers (Fig. 4c) were also slightly impacted by treatment with the high CO_2_ group being lower than either of the other two treatments for both lactate and glucose.

## Discussion

Here we show that the novel method of *in-situ* anaesthesia followed by artificial gill irrigation and caudal puncture can avoid the acute physical stress typically associated with ‘grab ‘n’ stab’ sampling. We also show that this method yields blood chemistry data comparable to that of the more technically challenging surgical cannulation of fish, with the added benefit of being applicable to fish typically too small to be cannulated.

This novel method has three main advantages over grab ‘n’ stab sampling. Firstly, it prevents the activation of the large mass of anaerobic white muscle (typically ~ 50% of body mass in fish^12^). Different sampling methodologies, and therefore differing levels of white muscle activation, have been shown to have significant impacts on the metabolites produced during struggling^41^ and therefore avoiding this release of metabolites is hugely beneficial. Secondly, it avoids stimulation of catecholamine release, which, whilst being famous in many teleostean fish for protecting red blood cell pH_i_ during the decline in extracellular pH (e.g., here in the stock tank/netted group), still deviates from the ‘resting’ acid–base status. Finally, with the gills fully submerged in the *in-situ* anaesthesia group, branchial respiratory gas exchange is maintained (which is impossible when fish are in air) preventing depletion of blood oxygen and further accumulation of CO_2_ (and potentially lactate if blood O_2_ declines sufficiently to promote anaerobic metabolism more widely than just white muscle). This avoids the rapid, acute hypoxia/hypercapnia and mixed respiratory/metabolic acidosis that most commonly occurs in the classic ‘grab ‘n’ stab’ method (here the stock tank/netted treatment). Fish in the isolated/netted group suffered a more moderate disturbance due to the shorter duration of the capture/netting process (and less struggling) but also their gills being irrigated during the sampling process itself. The *in-situ* anaesthesia method is therefore able to avoid many of the confounding effects of grab ‘n’ stab that can lead to misinterpretation of physiological responses.

The isolated/*in-situ* anaesthesia group had elevated cortisol compared to other groups and previous studies^32,42^, but it is worth noting that cortisol usually becomes elevated over a longer time frame than the sampling process here^43,44^. We interpret this elevation to have been caused by an insufficient recovery period after moving fish from the stock tanks to their isolation chambers (7 days in the isolated/*in-situ* anaesthesia fish versus 10 days in isolated/netted fish) rather than any stress during the anaesthesia and sampling process itself which can elevate cortisol in rainbow trout^32^. If keeping low cortisol levels is an experimental priority when sampling sufficient recovery time after handling should be factored in, and/or done in a manner where the effects of social isolation are minimised (e.g. in a clear tank submerged in a stock tank containing other conspecifics).

The isolation/*in-situ* anaesthesia method also clearly yields results comparable to those of cannulated animals under a range of conditions commonly used to experimentally perturb blood gas and acid–base status, in this case causing both acidosis and alkalosis for comparison with resting conditions^39,45–47^. The correlation observed between cannulation and caudal puncture concerning PO_2_ is weaker than other variables which is probably inevitable due to the proximity of the caudal artery and vein within the haemal arch^48^. Hence it is not possible to guarantee sampling either purely arterial blood (from the caudal artery) or purely venous blood (from the caudal vein) ever time, so samples will sometimes contain a mixture of venous and arterial blood. However, by measuring PO_2_ we can have a good idea of whether the sample is predominantly arterial or venous which can be useful in interpreting blood chemistry data. Interestingly, for the data shown in Fig. 3, most of the variability in blood PO_2_ from caudal puncture samples was in the control treatment. We are therefore tempted to conclude that this represents the researchers becoming more practised at hitting the artery rather than vein as the experiments progressed. Alternatively, we can speculate that the increased arterial blood pressure associated with stress-inducing scenarios (e.g. high environmental CO_2_ or alkaline water pH) may result in more arterial (i.e. more oxygenated) than venous blood being obtained in the sample.

We also validated the *in-situ* anaesthesia technique for use on fish one-tenth of the size of fish typically used for cannulation (~ 150 g). Naturally, the volume of blood that can be reliably withdrawn scales down with fish size. Also, for repeat sampling without termination this volume is dependent on local regulations for animal welfare. In the case of the UK, this cannot exceed 10% of the animal’s total blood volume in any 24-h period^49^ while in Canada this cannot exceed 0.1% of the fish’s body mass (~ 2.5% of blood volume) in cases where the fish will be recovered^50^. Careful consideration also needs to be taken regarding the speed of physiological processes in smaller fish that have higher mass-specific metabolic rates^51^. Here we aimed to sample at the peak of the alkaline tide in line with previous studies^38^ and while bicarbonate was elevated, blood pH was not significantly higher than the ‘control’ treatment. This may be influenced by the fact that gut transit times are faster in smaller fish^52^, and so we may have missed the peak of the alkaline tide. Care of course must be taken to ensure sample volumes are minimised to avoid haemodilution if serially sampling from the same fish over time^53–55^. Therefore if followed by a period of recovery where the fish is gill-irrigated in anaesthetic free water, this technique can be used to take serial blood samples. The potential for this technique to be applied to such small fish is a further reason why this technique can offer an important advance to the field of fish biology.

The acute nature of *in-situ* anaesthesia sampling also has the added benefit of minimising the likelihood of generating an immune challenge compared to surgical implantation of a cannula when taking serial samples. Samples taken via caudal puncture have significantly less impact on the phagocytic index, red and white cell count, and cortisol concentration than samples taken via indwelling catheters^31^ which obviously involve the implantation of a foreign body. Therefore, *in-situ* anaesthesia may provide a key tool in the field of fish immunology that avoids the immune challenge associated with cannulation without adding the confounding effects of handling stress that ‘grab ‘n’ stab’ sampling may introduce.

The technique presented herein also requires significantly less technical skill than cannulation, and if appropriate considerations are made, can be readily performed in the wild. In the case of highly social fish in laboratory experiments, it can also be adapted to simultaneously anaesthetise and sample whole groups of animals living in the same tank, assuming there are sufficient gill irrigation set-ups and personnel available to aid in sampling. For example, we have successfully sampled groups of 10 small rainbow trout at a time (body mass 8 to 16 g). There is also considerable flexibility in this technique and since the initial experimental work presented here, it has been adapted for a variety of fish species, of multiple different body forms and sizes including flatfish (e.g., European flounder, plaice) and round fish (e.g., rainbow trout, Atlantic salmon, European sea bass, lumpfish, barramundi).

Sufficient ventilatory flow of water during the gill irrigation process is clearly a requirement for normal blood gas and acid–base variables. When ventilatory water flow is experimentally reduced below typical in vivo rates this elevates arterial PCO_2_ and reduces pH and PO_2_, but at higher water flow rates the blood gases are hardly affected^34^. This is because at these high ventilatory flow rates the limiting factor becomes the branchial blood supply together with the gill diffusion capacity and chemical reaction velocities in the blood and water relevant to gas transfers^34^. All these limiting factors should be constant in our anaesthetised gill irrigation preparation, such that slightly over-ventilating the gills with water will have a negligible effect on blood pCO_2_ and acid–base status. This provides a very useful safety-margin when using this anaesthetised gill irrigation method. In practice we found that sufficient ventilatory flow was achieved when the inflow rate via the mouth was increased enough to cause the branchiostegal membrane to just separate from the body wall such that a small but distinct outflow of water via the operculae was just visible (see Methods for fuller description). However, under-ventilation clearly does risk generating higher pCO_2_ and lower pH and PO_2_ in the blood than the true in vivo state. In our experience the most common scenarios that created under-ventilation were (1) not all of the gill basket was fully submerged, and (2) insufficient water inflow via the mouth to ensure the operculae just open. However, with this knowledge these problems can easily be avoided.

One caveat worth noting is that some environmental disturbances in fish induce changes in ventilation that, at face value, would be expected to be important when trying to understand the holistic physiological responses in fish. For example, during exposure to hyperoxic water fish display reduced ventilation, elevated blood pCO_2_ and blood acidosis, followed by a slower compensatory rise in plasma bicarbonate to compensate and restore blood pH^40^. The gill irrigation method would therefore be less useful under such circumstances in establishing precise blood gas and acid–base responses unless able to precisely match ventilatory changes. However, in that previous hyperoxia study^40,54^, they found that the reduced gill ventilation itself had minimal influence on the initial blood hypercapnic acidosis observed. Instead, they concluded that branchial vasoconstriction caused internal diffusive and/or perfusive limitations which resulted in a rise in blood pCO_2_. This would suggest that the gill irrigation technique described here may even provide accurate blood acid–base data for experimental treatments that induce changes in external convection, i.e. gill ventilatory water flow.

## Supplementary Information

Supplementary Information 1.

Supplementary Information 2.

Supplementary Information 3.

Supplementary Information 4.

Supplementary Information 5.

Supplementary Information 6.

Supplementary Information 7.

Supplementary Information 8.

## Data Availability

All data presented herein are described as main effects and multiple comparisons. Details of descriptive statistics, variability, and effect sizes are given in supplementary materials. All data generated or analysed during this study are included in the supplementary information files associated with this publication.
