# Supplementary material for: A method for measuring meaningful physiological variables in fish blood without surgical cannulation
Source: Sci Rep. 2023 Jan 17;13:899. doi: 10.1038/s41598-023-28061-w (PMC9845352; doi:10.1038/s41598-023-28061-w)
Supplement: Supplementary file 1 — Supplementary Information 1. [file 41598_2023_28061_MOESM1_ESM.docx]

***Anaesthetic Choice***

We should point out that two different anaesthetics were used in the present study. Benzocaine was used in Experiments 1 and 3, whereas MS222 was used in Experiment 2. Both drugs have a similar mode of action and minimal impact on cardiovascular functions at the doses used^50^, so both can be used to provide sedation whilst maintaining a spontaneously ventilating fish. However, for future work we would recommend the use of benzocaine as this avoids complications caused by the influence of MS222 on the pH of the water, which in turn can dramatically alter the water pCO_2_ during the anaesthetic and sampling process. In Experiment 2 this problem was avoided by careful measurement and adjustment of pH (using NaOH) to precisely match the fish tank water (± 0.01 pH units) when preparing the working stock solution of MS222. This requires additional care and precise pH measurements and does add significant time to the process. It is also easy to overshoot the amount of NaOH required to reach the target pH and failure to exactly match this pH can result in surprisingly large differences in pCO_2_ in the water (and therefore impact the pCO_2_ and pH of the fish’s blood). However, if these steps are performed accurately, the results of Experiment 2 show that MS222 is still a valid anaesthetic for determination of accurate acid-base data.
