# Supplementary material for: A method for measuring meaningful physiological variables in fish blood without surgical cannulation
Source: Sci Rep. 2023 Jan 17;13:899. doi: 10.1038/s41598-023-28061-w (PMC9845352; doi:10.1038/s41598-023-28061-w)
Supplement: Supplementary file 3 — Supplementary Information 3. [file 41598_2023_28061_MOESM3_ESM.docx]

| Experiment | Endpoint | Treatment | Number of Exclusions | Reason | Final n |
| --- | --- | --- | --- | --- | --- |
| Experiment 1 | pO_2_ (kPa) | Stock Tank/Netted | 0 | N/A | 8 |
|  |  | Isolated/Netted | 0 | N/A | 6 |
|  |  | Isolated/ *in situ* anaesthesia | 0 | N/A | 6 |
|  | pH_e_ | Stock Tank/ Netted | 0 | N/A | 8 |
|  |  | Isolated/Netted | 0 | N/A | 6 |
|  |  | Isolated/ *in situ* anaesthesia | 0 | N/A | 6 |
|  | pHi | Stock Tank/ Netted | 0 | N/A | 8 |
|  |  | Isolated/Netted | 0 | N/A | 6 |
|  |  | Isolated/ *in situ* anaesthesia | 0 | N/A | 6 |
|  | PCO_2_ | Stock Tank/ Netted | 0 | N/A | 8 |
|  |  | Isolated/Netted | 0 | N/A | 6 |
|  |  | Isolated/ *in situ* anaesthesia | 0 | N/A | 6 |
|  | HCO_3_^-^ | Stock Tank/ Netted | 0 | N/A | 8 |
|  |  | Isolated/Netted | 0 | N/A | 6 |
|  |  | Isolated/ *in situ* anaesthesia | 0 | N/A | 6 |
|  | [H^+^] | Stock Tank/ Netted | 0 | N/A | 8 |
|  |  | Isolated/Netted | 0 | N/A | 6 |
|  |  | Isolated/ *in situ* anaesthesia | 0 | N/A | 6 |
|  | Hct | Stock Tank/ Netted | 0 | N/A | 8 |
|  |  | Isolated/Netted | 0 | N/A | 6 |
|  |  | Isolated/ *in situ* anaesthesia | 0 | N/A | 6 |
|  | [Hb] | Stock Tank/ Netted | 0 | N/A | 8 |
|  |  | Isolated/Netted | 0 | N/A | 6 |
|  |  | Isolated/ *in situ* anaesthesia | 0 | N/A | 6 |
|  | MCHC | Stock Tank/ Netted | 1 | Outside 95 % CI | 7 |
|  |  | Isolated/Netted | 0 | N/A | 6 |
|  |  | Isolated/ *in situ* anaesthesia | 0 | N/A | 6 |
|  | Lactate | Stock Tank/ Netted | 0 | N/A | 8 |
|  |  | Isolated/Netted | 0 | N/A | 6 |
|  |  | Isolated/ *in situ* anaesthesia | 0 | N/A | 6 |
|  | Glucose | Stock Tank/ Netted | 0 | N/A | 8 |
|  |  | Isolated/Netted | 0 | N/A | 6 |
|  |  | Isolated/ *in situ* anaesthesia | 0 | N/A | 6 |
|  | Cortisol | Stock Tank/ Netted | 1 | Outside 95 % CI | 7 |
|  |  | Isolated/Netted | 1 | Outside 95 % CI | 5 |
|  |  | Isolated/ *in situ* anaesthesia | 0 | N/A | 6 |
|  | Adrenaline | Stock Tank/ Netted | 2 | Samples lost during analysis | 6 |
|  |  | Isolated/Netted | 3 | Values below detection threshold of assay | 3 |
|  |  | Isolated/ *in situ* anaesthesia | 0 | N/A | 6 |
|  | Norepinephrine | Stock Tank/ Netted | 0 | N/A | 8 |
|  |  | Isolated/Netted | 3 | Values below detection threshold of assay | 3 |
|  |  | Isolated/ *in situ* anaesthesia | 1 | Outside 95 % CI | 5 |
| Experiment 2 | pO_2_ | Control | 0 | N/A | 5 |
|  |  | High CO_2_ | 0 | N/A | 5 |
|  |  | High pH | 0 | N/A | 5 |
|  | pH_e_ | Control | 0 | N/A | 5 |
|  |  | High CO_2_ | 0 | N/A | 5 |
|  |  | High pH | 0 | N/A | 5 |
|  | HCO_3_^-^ | Control | 0 | N/A | 5 |
|  |  | High CO_2_ | 0 | N/A | 5 |
|  |  | High pH | 0 | N/A | 5 |
|  | pCO_2_ | Control | 0 | N/A | 5 |
|  |  | High CO_2_ | 0 | N/A | 5 |
|  |  | High pH | 0 | N/A | 5 |
| Experiment 3 | pO_2_ | Control | 0 | N/A | 10 |
|  |  | High CO_2_ | 0 | N/A | 10 |
|  |  | Fed | 0 | N/A | 10 |
|  | pH | Control | 2 | Outside 95 % CI | 8 |
|  |  | High CO_2_ | 0 | N/A | 10 |
|  |  | Fed | 0 | N/A | 10 |
|  | pCO_2_ | Control | 2 | Outside 95 % CI | 8 |
|  |  | High CO_2_ | 0 | N/A | 10 |
|  |  | Fed | 0 | N/A | 10 |
|  | HCO_3_^-^ | Control | 2 | Outside 95 % CI | 8 |
|  |  | High CO_2_ | 0 | N/A | 10 |
|  |  | Fed | 0 | N/A | 10 |
|  | [H^+^] | Control | 1 | Outside 95 % CI | 9 |
|  |  | High CO_2_ | 0 | N/A | 10 |
|  |  | Fed | 0 | N/A | 10 |
|  | [Hb] | Control | 0 | N/A | 10 |
|  |  | High CO_2_ | 0 | N/A | 10 |
|  |  | Fed | 0 | N/A | 10 |
|  | Hct | Control | 1 | Sample lost during processing | 9 |
|  |  | High CO_2_ | 0 | N/A | 10 |
|  |  | Fed | 0 | N/A | 10 |
|  | MCHC | Control | 2 | 1 x sample lost during processing  1 sample outside 95 % CI | 8 |
|  |  | High CO_2_ | 0 | N/A | 10 |
|  |  | Fed | 0 | N/A | 10 |
|  | Lactate | Control | 0 | N/A | 10 |
|  |  | High CO_2_ | 0 | N/A | 10 |
|  |  | Fed | 0 | N/A | 10 |
|  | Glucose | Control | 0 | N/A | 10 |
|  |  | High CO_2_ | 0 | N/A | 10 |
|  |  | Fed | 0 | N/A | 10 |
