# Supplementary figures and images for: A method for measuring meaningful physiological variables in fish blood without surgical cannulation
Source: Sci Rep. 2023 Jan 17;13:899. doi: 10.1038/s41598-023-28061-w (PMC9845352; doi:10.1038/s41598-023-28061-w)

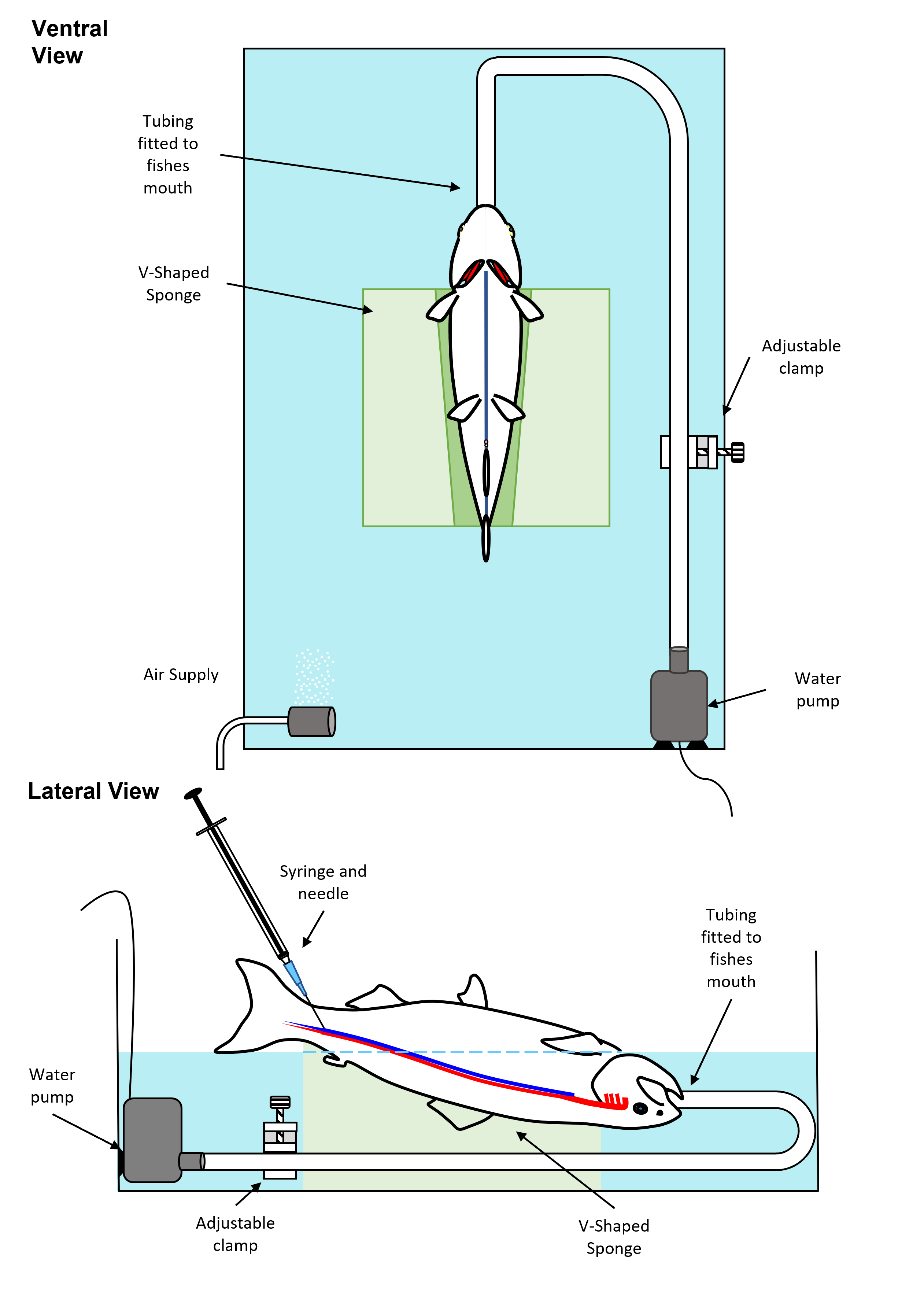

Supplement: Supplementary file 4 — Supplementary Information 4. [file 41598_2023_28061_MOESM4_ESM.png]
